# Efficient and accurate tiller counting of hand-collected samples using images of straw bundles

**DOI:** 10.1016/j.mex.2026.103837

**Published:** 2026-02-20

**Authors:** Gomathi Saravanan, Annett Latsch, Matthias Hatt, Thomas Anken, Ralph L. Stoop

**Affiliations:** Digital Production, Agroscope, 8356 Ettenhausen, Switzerland

**Keywords:** Plant phenotyping, Crop density, Hand-derived crop samples, Computer vision, Hough detection, Yield estimation

## Abstract

We present a novel method for accurately counting winter wheat tillers based on RGB images from hand-collected samples. An efficient sample preparation method assembles wheat tillers into bundles from which individual tillers are robustly detected automatically, using classical image analysis. A custom-made user interface (‘TillerCounter’ program) allows adjusting the automatic detections interactively, which leads to highly accurate tiller counts comparable to the ground truth obtained by manual counting.

The key contributions of our work include:

1. An efficient method for imaging straw tillers based on bundle assembly.
2. An extensive study of the obtained image quality and comparison with the ground truth data from manual counting.
3. Demonstration of the approach’s high accuracy using correlation analysis (Pearson correlation coefficient *R* = 0.973 compared to ground truth) and error analysis (root mean squared relative errors below 5 %).

## Graphical abstract

**Figure:**
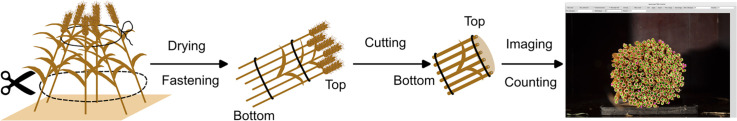

Specifications table

**Table:** 

**Subject area**	Agricultural and Biological Sciences
**More specific subject area**	*Plant phenotyping*
**Name of your method**	*AGS TillerCounting*
**Name and reference of original method**	*Hough, Paul V. C. Method and means for recognizing complex patterns. US3069654A, filed 25. März 1960, and issued 18. Dezember 1962.*
**Resource availability**	The source code of the TillerCounter GUI is given at https://github.com/agroscope-ch/TillerCounterGui. Original data is given at Zenodo repository 10.5281/zenodo.14446564

## Background

To meet the global demand for food production in the future, innovative concepts and novel techniques are needed to intensify sustainable crop production. Plant samples collected by hand (‘hand samples’) are an important subject of study to evaluate the effectiveness of such novel measures. In particular in breeding experiments and field plot experiments, hand samples often serve as a starting point for experimental analysis in the context of plant phenotyping. A hand sample is harvested from a well-defined, small area of a field and thus consists of a collection of plants that originally grew in close proximity to each other. One crucial aspect of a hand sample is the total plant count per unit area, which is particularly interesting when studying crops such as winter wheat. In this case, combined with the thousand kernel weight and the grain weight, the number of tillers per surface allows e.g., the determination of the number of kernels per ear, which is an important yield parameter.

When high accuracy is needed, counting the number of tillers of a hand sample manually remains the gold standard, provided that it is done very carefully. However, manual counting is extremely time consuming, in particular due to the difficulty of separating individual tillers from a hand sample, as they are often entangled. In addition, errors that occur during counting are difficult to assess at a later date since hand samples cannot be stored efficiently in large quantities, making data traceability difficult.

To quantify the tiller density automatically, novel approaches have recently been suggested in the literature. Most of these methods focus on large scale, high throughput analysis of extended fields. These methods are based either on RGB photography [[1], [2], [3], [4], [5], [6], [7], [8], [9]] or by alternative sensing devices such as radiometric [10,11], radiometric combined with ultrasonic [12,13] or LiDAR sensors [14,15]. Since these methods can be applied without destructive harvesting, they enable monitoring of tiller density dynamics throughout the season to estimate e.g., yield. The reported relative errors are usually in the range of 10–30 %, which is sufficient for yield estimation. Advances in deep learning-based methods have recently lowered this limit even further [7]. Zang et al. demonstrated relative errors of only 3.51 % when comparing deep learning-based counts with image annotations [6]. However, deep learning-based approaches may not always generalize well to new data, such as wheat fields with markedly different visual appearances. In addition, methods based on in-field images can still suffer from occlusion and are not optimized for hand samples, which require highly accurate counts, in particular for scientific studies where hand samples form the experimental unit. New, complementary methods are therefore needed for objective, transparent, and accurate counting of plant tillers in hand samples collected from field plots. Ideally, these methods can bridge the currently existing gap between the accuracy of hand counting and the efficiency of remote sensing techniques for highly accurate count estimation of hand samples.

In this work, we present a simple yet robust sample preparation process that allows a highly accurate and efficient counting of tillers from winter wheat. Our method is based on assembling straw tillers into bundles and cutting both sides to provide a clean cross section of the bundle. Individual tillers are easily identified from RGB images taken from the bundle’s cross section under controlled lighting conditions. Using a custom ‘TillerCounter’ program with a convenient user interface, automatic detections from a Hough circle detector can be adjusted by the user in a semi-automated fashion, significantly reducing the overall counting time. We validate our approach on a collection of bundles from 13 distinct sites, demonstrating our method’s robustness.

## Method details

### Sample collection

Sample collection and preparation were carried out within the ‘Smart-N’ project, which investigates the impact of site-specific fertilization on winter wheat yield and nitrogen surpluses [16]. The study included fields from seven different farms in Eastern Switzerland and recorded different wheat data for the years 2023–2024 from a total of 22 fields.

**Fig. 1:**
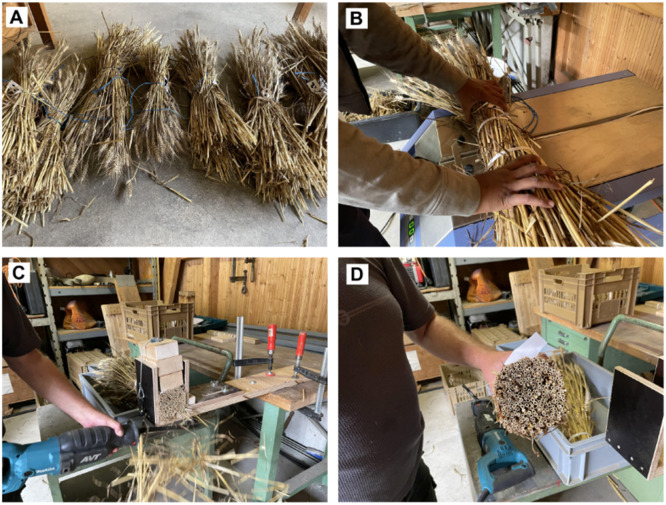
Images of the sample preparation process. A: Bundles after in-field cutting. B: After drying, the samples are fixed using a parcel strapping machine. C: Each side of the bundle is cut using an electric saw. During cutting, the samples are fixed using a wooden clamp. D: The resulting cross-section.

At each site, patches of 0.5 m width (equivalent to four seeding lines) and length of 1 m, leading to a patch area of 0.5 m^2^, were selected for harvesting. The typical number of patches utilized per site was 30, which yielded approximately 330 sample bundles per year. The tillers of each patch were cut at a height of approximately 12 cm above ground level using an electric hedge trimmer. Following the cutting process, all samples from a single patch were carefully bundled together with a rope and individually labeled before being transported to the storage room (Fig. 1A). The samples were dried under ambient conditions to prevent mold formation.

### Sample preparation

In a previous year excluded from this work (2022), tillers were manually counted, which proved to be a labor-intensive and time-consuming process. Counting by hand strongly depends on the operator’s focus and concentration, suffers mainly from exhaustion or interruption, and lacks reproducibility once the samples are discarded. In this work, samples from years 2023 and 2024 were recorded by standard photography, which allowed us to reproduce the counting results at any time. To facilitate counting, each bundle was fastened tightly, using a standard parcel strapping machine (Orgapack, Dietikon, Switzerland), as shown in Fig. 1B. Thereby, robust cables were attached at three different locations along the bundle. Special attention was devoted to determining the optimal strapping force: an insufficiently tight strap may cause individual straw tillers to dislodge from the bundle, while too tight strapping can squeeze the tillers, complicating accurate counting. After initial adjustment, all bundles were strapped with the same force to speed up the bundling process. On each side along the bundles’ longitudinal orientation, a clean cross-sectional area was achieved via cutting with an electric saw (JR3060T Makita, Aichi, Japan) using a blade intended for fiber insulation (S1213AWP, Bosch, Stuttgart, Germany). The type and properties of the blade crucially influenced the quality of the cutting section, and the selected blade minimized the frayed edges. Residuals from the top cut containing the ears of the wheat samples were collected and processed separately. Residuals from the bottom part were discarded. During cutting, the bundle was tightly fixed using a custom-made wooden clamp (Fig. 1C). A tight fixation prevented fraying of the edges of the individual tillers. A clean vertical cut also ensured a flat cross-section, which was essential to have all tiller edges with a similar distance to the camera and ensured high visibility of each tiller’s cross section (Fig. 1D).

**Fig. 2:**
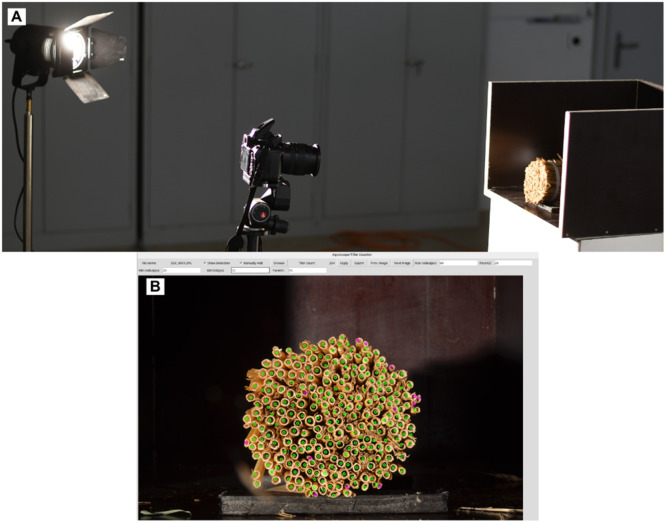
A: Imaging setup for the tiller samples. B: Graphical user interface of the TillerCounter program.

After cutting the samples, each bundle was positioned in a dark wooden box with one of the cutting surfaces aligned with the open side of the box. The use of a standard photography lamp (Kaiser studiolight 1000, Kaiser Fototechnik, Buchen, Germany) with an incidence angle of approximately 45° with respect to the bundle surface resulted in an increased contrast between the tiller edges and the void inner part of the tiller while maintaining constant illumination of the samples (Fig. 2A). Images were captured using a standard SLR camera (Nikon D5100, Nikon, Minato, Japan) in manual mode with consistent aperture settings (F-number F22) and ISO-100, using autofocus for consistent focal positioning. After initial trials with different exposure times in 2023, a constant exposure time of 0.17 s was used for all images in 2024. A single image was obtained from each side of the bundle (front and back) to assess the reproducibility of the counts from each side for the same bundle.

### Hough transform-based TillerCounter

To analyze each bundle, we developed a Python-based counter tool, named ‘TillerCounter’, which employs a classical circle detection method based on the Hough transform to identify tillers [17]. The tool provides a basic graphical user interface (GUI) for the selection of images and the application of the Hough transform for the detection of circular structures corresponding to tillers (Fig. 2B). Prior to the application of the Hough transform, the image is converted to grayscale, and the noise is reduced by Gaussian blurring with a median filter of 5 × 5 pixels. These pre-processing steps serve to minimize false-positive detections and are commonly applied prior to shape detection using Hough transforms. Automated counting is based on OpenCV’s implementation of Hough-transform (function: cv2.HoughCircles(.)) using the HOUGH_GRADIENT detection method to identify circular structures in the image [18,19]. The function requires two parameters specific to the selected detection method ($p_{1,2}$). Further, the minimum distance between the two shapes ($d_{min}$) and the minimum ($r_{min}$) and maximum ($r_{max}$) radii of the targeted circular structures are given. The values of all parameters can be directly adjusted in our GUI. However, the parameters mostly remained approximately constant, since most bundle images share a similar scale, as the distance between the camera and bundle cutting surface remains constant during image acquisition. Changes in tiller sizes, however, are certainly possible, particularly between bundles from different sites or treatments. In such cases, adjusting the parameters can considerably improve the number of automatically detected tillers. The detected circles are directly displayed in the image, allowing the user to validate or correct the automated tiller detection. To do so, the user may add previously undetected tillers by left-clicking at the corresponding location or remove a falsely detected “tiller” by right-clicking close to the corresponding annotation.

The TillerCounter tool was implemented using Python (version 3.8 or higher [20]) and the tkinter package [21] for the GUI (see data availability statement). Additional standard Python libraries, including NumPy [22] for numerical operations, OpenCV-python [23] for image processing, and pillow [24] for image manipulation, were employed. The program was tested on Ubuntu 20.04 [25] and higher, ensuring compatibility with Python 3.8 or later.

### Data analysis and validation

Images from wheat bundles from two consecutive years (2023: 331 bundles and 2024: 318 bundles) were analyzed for the present study. The original data consisted of two images (top and bottom cutting surfaces) per bundle (2023: 662 images and 2024: 636 images). All images were analyzed using the *TillerCounter* program: For each image (two per bundle), the final “**Hough + adjusted count**” resulted from the number of automatically detected tillers (“**Hough count”**) via correction by the number of the manually added and removed counts, that is, ***“Hough + adjusted count” = “Hough count”*** *+ “manually added” – “manually removed”*. Results from the Hough predictions were controlled and corrected manually for each image.

To validate the approach, pure manual counting from bundle images by human annotators (“**annotation count”**) was carried out for 124 images of a validation subset comprising 62 bundles. Similar to the Hough + adjusted counts, annotation counts were determined for each side of the bundle. Annotation counts were used to study the effect of using the semi-automated approach with the *TillerCounter* program, as it might introduce some bias. The annotation counts quantified how well tillers could be detected by eye and whether occlusion of tillers or remaining plant leaves could lead to false negatives or false positives, respectively. Under the assumption that human annotators are superior to any (semi-)automated counting process, the annotation counts served as an optimal baseline for image-based methods. To obtain annotation counts, the individual tillers were manually marked by color (using the image processing software GIMP [26]) to avoid double counts. Additional hand counts of tillers (“**ground truth count**”) were carried out for the bundles of the validation set. Thereby, each bundle was disassembled, and all tillers were carefully counted by hand. The hand counts served as the ground truth in the remainder of this work.

## Evaluation metrics

To quantify the potential of our approach and compare annotation counts with Hough and Hough + adjusted counts and ground truth counts, different metrics were used. To demonstrate how well the measured quantity related to the ground truth, we used the Pearson correlation coefficient R, defined by standardizing the covariance $Cov(y_{i},{\hat{y}}_{i})$ between ground truth $y_{i}\;$and estimation ${\hat{y}}_{i}$:

$$R=\frac{Cov\left(y_{i},{\hat{y}}_{i}\right)}{\sqrt{Var\left(y_{i}\right)Var\left({\hat{y}}_{i}\right)}}$$

Here, $Var(y_{i})$, $Var({\hat{y}}_{i})$ is the variance of the ground truth and estimation, respectively. To quantify the errors, we used the mean-squared error (MSE) and root mean square error (RMSE), where

$$MSE=\frac{1}{N}\sum_{i=0}^{N-1}{\left({\hat{y}}_{i}-y_{i}\right)}^{2}$$

and $RMSE=\sqrt{MSE}$. Furthermore, the relative error in % was calculated using $\frac{{\hat{y}}_{i}-y_{i}}{y_{i}}\;\times \;100\;$ for each counting method and image *i*. To determine the overall accuracy of one of the investigated methods (Hough count, Hough + adjusted count and annotation count), we used the root mean square relative error (RMSRE) calculated over all images *i*:

$$RMSRE=\sqrt{\frac{1}{N}\sum_{i=0}^{N-1}{\left(\frac{{\hat{y}}_{i}-y_{i}}{y_{i}}\;\times \;100\%\right)}^{2}}$$

## Method validation

**Fig. 3:**
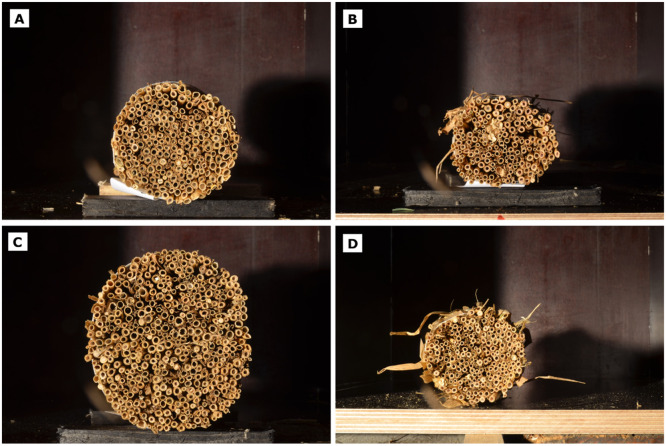
Exemplary photographs showing large varieties of bundles and individual tillers. For some samples, residual plant remainders on the surface of the bundle were removed prior to imaging (A and C), as they can contribute to false detections.

Photographs of the top side of four different tiller bundles after cutting are shown in Fig. 3. For their corresponding bottom side, see Figure S1 of the supplementary material (SM). Depending on the site of origin and in-field location, the cutting sections varied considerably in total size and shape, as well as individual tillers’ appearance. Whereas some bundles presented tillers as relatively closed-packed (Fig. 3A), others depicted holes or even residuals from the ears (Fig. 3B). Size differences were also present, from large tillers in big bundles (Fig. 3C) to tiny tillers in small bundles (Fig. 3D). Over all samples, we found that the number of tillers per bundle varied between 100 and 460, with most bundles being around 250 (Figure S2 in the SM).

**Fig. 4:**
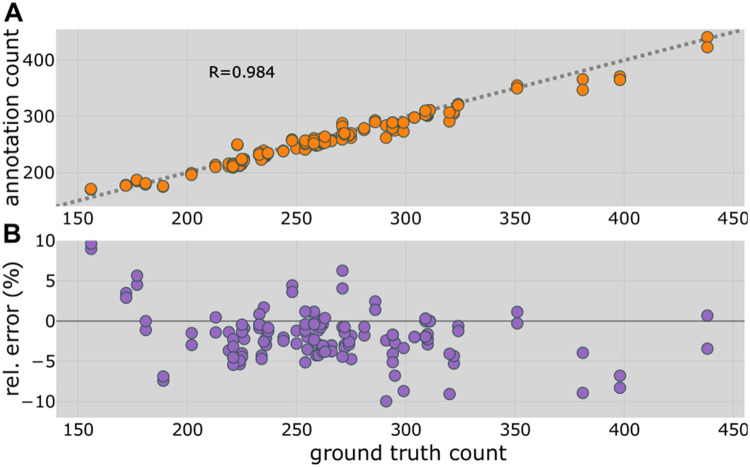
Comparison of counting tillers from bundle images by eye (annotation count) and the corresponding ground truth count (determined by hand counting). A. Annotation count versus ground truth count for both sides of 62 validation bundles. *R* = 0.984 shows a high linear correlation. $\mathrm{RMS}E_{\mathrm{annotation}\;\mathrm{count}}=10.7$ tillers per bundle or 21.4 tillers/m^2^. B. Relative error of the annotation count versus ground truth count.

Given the large variety in bundle appearances, the question emerged of how well the acquired images could allow for identifying and counting tillers. To answer this question, the annotation counts were compared to their ground truth data for the bundles of the validation set (62 bundles, 124 images) in Fig. 4A. A highly linear correlation, expressed by *R* = 0.984 over a wide range of bundle sizes, was observed. Given that each cutting side (top/bottom) of a bundle contributed to an individual data point in Fig. 4A, an important question was how similar the annotation counts of the two sides of the same bundle were. A very high correlation (*R* = 0.991) was observed between the two sides, indicating good agreement between annotation counts derived from the top and bottom sides of the bundle, see Figure S3 of the SM. The relative error versus the ground truth count is shown in Fig. 4B. Note that the two sides of each bundle are included here as individual data points, similar to Fig. 4A. Maximum deviations of < 10 % for the smallest and largest bundles were observed and, generally, a slight underestimation of tiller number. The obtained *RMSRE_annotation count_* = 3.97 % demonstrates the high quality of the bundling process for tiller counting.

**Fig. 5:**
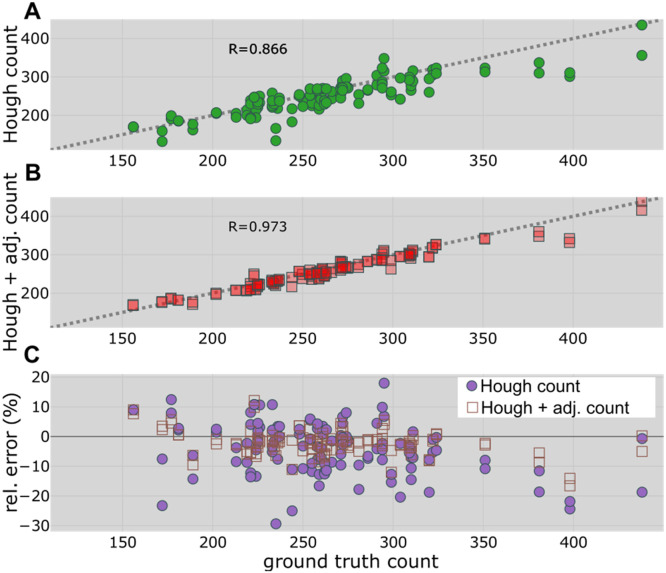
Comparison of TillerCounter-based counting with ground truth (determined by hand counting) for 62 validation bundles. A. Automatically detected Hough counts versus ground truth counts. B. Manually corrected TillerCounter counts (Hough + adjusted counts) versus ground truth counts. C. Relative error versus ground truth counts for Hough counts (filled circles) and Hough + adjusted counts (open squares). $\mathrm{RMS}E_{\mathrm{Hough}\;\mathrm{count}}=28.8\;$ tillers per bundle or 57.6 tillers/m^2^ and $\mathrm{RMS}E_{\mathrm{Hough}+\mathrm{adj}.\;\mathrm{count}}=13.7$ tillers per bundle or 27.4 tillers/m^2^.

In a fully automated fashion, the uncorrected Hough count could be used directly as an estimate of the number of tillers in the bundle. The resulting scatter plot (Hough counts versus ground truth counts) is shown in Fig. 5A with *R* = 0.866. Allowing users to correct the Hough detections using the proposed semi-automatic approach (Hough + adjusted count) improved the correlation with the ground truth counts (R = 0.973), as shown in Fig. 5B. The relative error of Hough counts (violet filled circles) and Hough + adjusted counts (brown open squares) versus ground truth counts are shown in Fig. 5C. Whereas we observed relative errors < 30 % for the automatic Hough counts, the relative errors remained mostly < 10 % after corrections using the TillerCounter program. The resulting root mean square relative errors were given by *RMSRE_Hough count_* = 10.26 % and *RMSRE_Hough__+__adj. count_* = 4.74 %.

Compared to the annotation count (R = 0.984), our semi-automated approach sacrificed some accuracy (R = 0.973) for higher processing speed. Since both annotation counts and Hough + adjusted counts were based on the same images, in principle, both methods could achieve the same accuracy, given that a human counter could correct all falsely detected/undetected tillers. However, we used the TillerCounter tool to efficiently examine our complete dataset, which was much faster than complete manual counting by eye using bundle images or by hand using the bundle directly. Hence, the decrease in accuracy must be related to the time savings achieved.

Although the exact amount of time saved was hard to measure and strongly depended on the person performing the counting, we estimate that the presented semi-automatic approach reduced the time from roughly 3.5 min for purely counting by eye (annotation count) to roughly 1.5–2 min using the TillerCounter program (Hough + adjusted count). The time needed to count wheat tillers manually (ground truth count) was about 4–8 min per bundle, mainly due to the difficulty of separating individual tillers from the bundle, as they were typically entangled. Accordingly, our approach reduced the total counting time by a factor of 2–4, as strapping and cutting did not require substantial time (< 1min/bundle).

Counting by hand is usually error-prone, particularly when dealing with large quantities of bundles that need to be processed in time for further processing, such as protein content analysis. Our approach separates wheat ears from the tillers at an early stage, entangling counting from other physical and chemical wheat analysis. This allows for more flexible planning and better utilization of critical infrastructure such as threshing machines. Note that our results are based on bundles from dense fields with densities of about 300–800 tillers/m^2^. For some use cases, a mean relative error of ≈10% might be sufficient, as achieved with the fully automatic Hough detection method. When highest accuracies are needed, manual adjustments are still required. In this case, our semi-automatic TillerCounter tool provides a simple yet effective method.

## Limitations

To achieve high quality bundles, our assembly method requires crop tillers with sufficient mechanical stability.

To improve the results of automated detection, it was necessary to properly adjust the parameters of the cv2.HoughCircle() function. However, we generally kept the parameters constant unless there were significant detection errors, for example, when the tiller diameters differed greatly between different sites. There were two reasons for our approach. First, tuning the parameters required some experience with image analysis, which a labeling/counting person might not have. Second, parameter tuning also required time that could alternatively be spent on direct manual corrections.

**Fig. 6:**
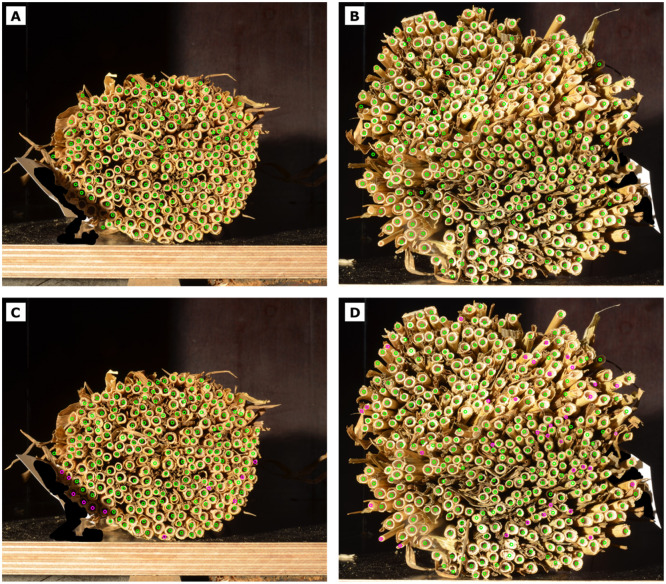
Two bundles (left/right) from 2023 with Hough counts (top) and Hough + adjusted counts (bottom) using constant Hough parameters of r_min_ =20, r_max_ =54, p_1_=95, p_2_=30, d_min_=50. Left bundle: $n_{\mathrm{Hough}}=243$, $n_{\mathrm{added}}=11$, $n_{\mathrm{removed}}=11$ and $n_{\mathrm{Hough}+\mathrm{adj}.}=243$. Right bundle: $n_{\mathrm{Hough}}=392$, $n_{\mathrm{added}}=36$, $n_{\mathrm{removed}}=43$ and $n_{\mathrm{Hough}+\mathrm{adj}.}=385$. Detections from Hough method are highlighted by the green circles, manual corrections by the pink circles.

An example of Hough detections and corresponding manual corrections of two bundles from different sites is shown in Fig. 6. For both bundles, the same Hough parameters were used. While the bundle shown on the left side required only minor corrections in the range of <5 % of the total count, the bundle on the right side required substantially more corrections, namely about 12 % of the total count.

**Fig. 7:**
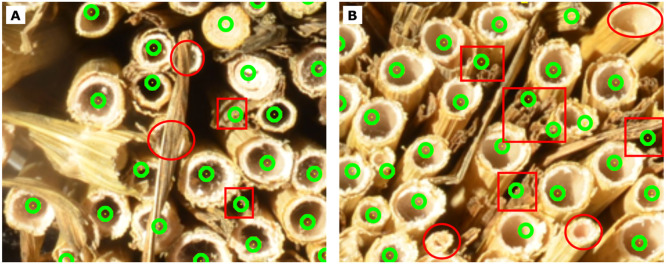
Failure cases, exemplified by zoomed regions from the bundle shown in Fig. 6B. Undetected tillers (false negatives) are highlighted by red circles, leaves and gaps misclassified as tillers (false positives) are highlighted by red rectangles.

Two main aspects affected the quality of Hough detections of the bundle in Fig. 6B, D. First, the bundle contained many leaves, which could lead to occlusion, as highlighted in Fig. 7A. Second, while the tillers of the bundle shown in A, C were all similar in size, the tillers of the bundle shown in B, D varied greatly in size and shape. Capturing tillers of very different sizes required small $r_{min}$ and large $r_{max}$ values. However, a large radius range also made false positive detections more likely, because Hough’s method detects any circular structure that fits within the range, as shown in Fig. 7B. These false positives were mostly due to folded plant leaves that formed circular structures in the gap between the wheat tillers and were not always easy to correct manually. Although false positives could be reduced by increasing $d_{min}$, this could lead to an increased number of false negatives, in particular in regions with small-diameter tillers. It thus remained challenging to choose the values of $r_{min}$ and $r_{max}$ and $d_{min}$ properly to avoid misdetections, making manual corrections necessary, in particular for bundles with widely varying tiller diameters.

Learning-based methods have the potential to further automate the counting of tillers from images created by the proposed bundle process, given that a sufficiently large fraction of images is accurately annotated. However, it remains to be seen how robust a deep learning model can detect tillers from images derived from the suggested process. This offers a promising direction for future research.

## Ethics statements

None.

## CRediT author statement

**Gomathi Saravanan**: Methodology, Validation, Writing - review & editing. **Annett Latsch**: Conceptualization, Methodology, Writing - review & editing. **Matthias Hatt**: Methodology, Investigation. **Thomas Anken**: Conceptualization, Methodology, Writing - review & editing. **Ralph L. Stoop**: Supervision, Software, Writing - original draft.

## Declaration of generative AI and AI-assisted technologies in the writing process

During the preparation of this work the author(s) used DeepL in order to refine and reformulate sentences. After using this tool/service, the author(s) reviewed and edited the content as needed and take(s) full responsibility for the content of the publication.

## Declaration of competing interest

The authors declare that they have no known competing financial interests or personal relationships that could have appeared to influence the work reported in this paper.

## Data Availability

The source code of the TillerCounter GUI is given at https://github.com/agroscope-ch/TillerCounterGui. Original data is given at Zenodo repository: 10.5281/zenodo.14446564

## Appendix Supplementary materials

Image, application 1
