# Supplementary material for: Efficient and accurate tiller counting of hand-collected samples using images of straw bundles
Source: MethodsX. 2026 Feb 20;16:103837. doi: 10.1016/j.mex.2026.103837 (PMC12964300; doi:10.1016/j.mex.2026.103837)
Supplement: Supplementary file 1 [file mmc1.docx]

**Supplementary Material**

## Differences Between Top and Bottom Side

Figure S1 shows the photographs of the bottom part of the same four bundles, whose top view is also presented in Figure 3 of the original manuscript. Individual counts from both sides of the bundle provide some repeatability.


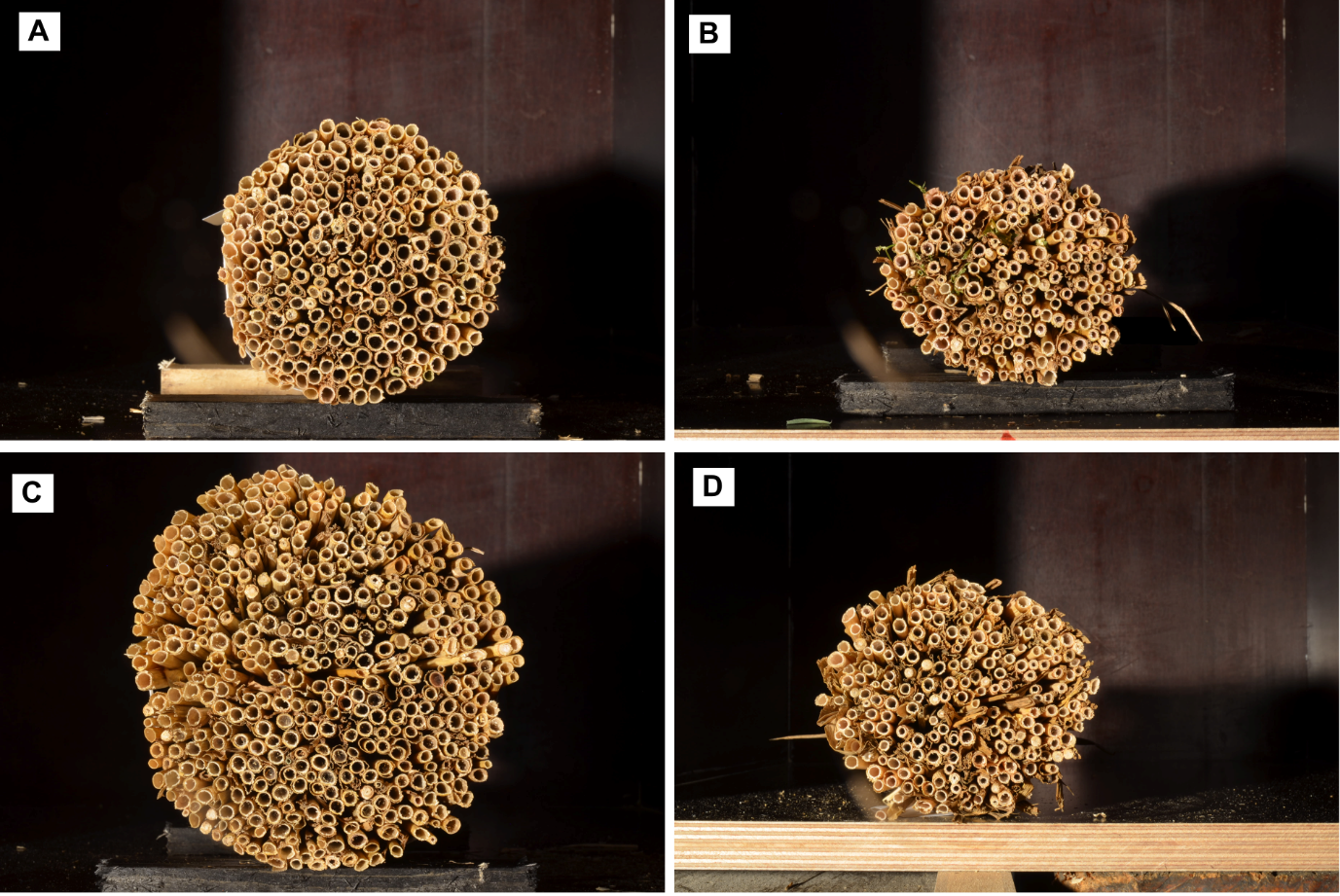


*Figure S1 – Four sample images of bundle cutting areas. Images correspond to the bottom side of the bundles shown in Figure 3 of the original manuscript.*

## Comparison Hough Count vs Hough + Adjusted Count

As discussed in the manuscript, a fully automatic counting of a bundle’s wheat tillers using Hough transform is generally not accurate enough and results in relative errors of around 10%. Figure S2 shows a comparison between Hough counts (orange color) and the manually corrected Hough + adjusted counts (blue color) by human labelers.


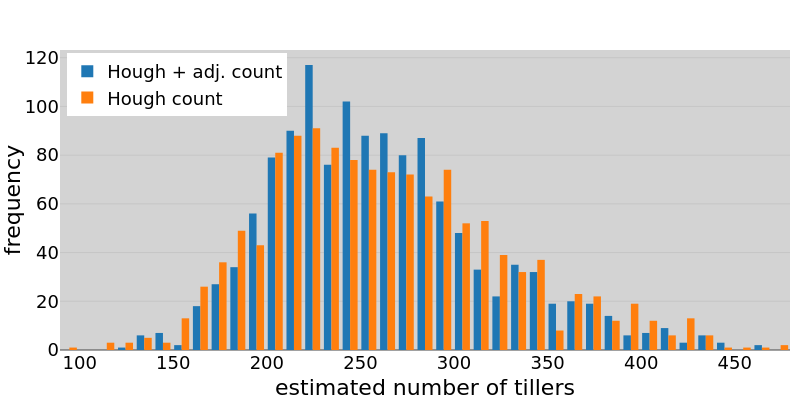


*Figure S2 – Histogram of Hough and Hough + adjusted counts for all bundles.*

## Correlation of Annotation Counts Derived from Top versus Bottom Side

Figure S3 shows the annotation count derived from the bottom side vs annotation count derived from the top side. High correlation is found.


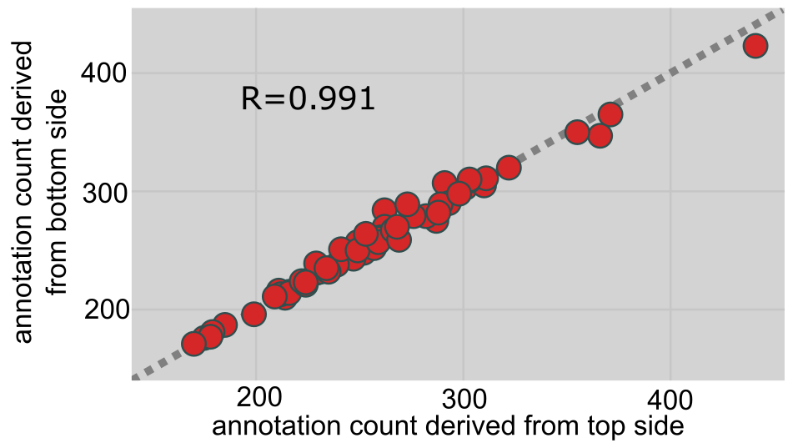


*Figure S3 Annotation counts (counts from bundle images by eye) derived from the bottom side vs. annotation counts derived from the top side.*
